# Integrative microRNA and gene profiling data analysis reveals novel biomarkers and mechanisms for lung cancer

**DOI:** 10.18632/oncotarget.7264

**Published:** 2016-02-08

**Authors:** Ling Hu, Junmei Ai, Hui Long, Weijun Liu, Xiaomei Wang, Yi Zuo, Yan Li, Qingming Wu, Youping Deng

**Affiliations:** ^1^ Department of Anesthesiology, Tianyou Hospital, Wuhan University of Science and Technology, Wuhan, China; ^2^ Department of Internal Medicine and Biochemistry, Rush University Medical Center, Chicago, IL, USA; ^3^ Department of Gastroenterology, Tianyou Hospital, Wuhan University of Science and Technology, Wuhan, China; ^4^ Department of Orthopedics, Pu Ai Hospital, Affiliated to Tongji Medical College, Huazhong University of Science and Technology, Wuhan, China; ^5^ Department of Biological Science and Technology, Wuhan Bioengineering Institute, Wuhan, China; ^6^ Department of Orthopedic, Tianyou Hospital, Wuhan University of Science and Technology, Wuhan, China; ^7^ Medical College, Wuhan University of Science and Technology, Wuhan, China

**Keywords:** microRNAs, lung cancer, meta-analysis, target gene, biomarker

## Abstract

**Background:**

Studies on the accuracy of microRNAs (miRNAs) in diagnosing non-small cell lung cancer (NSCLC) have still controversial. Therefore, we conduct to systematically identify miRNAs related to NSCLC, and their target genes expression changes using microarray data sets.

**Methods:**

We screened out five miRNAs and six genes microarray data sets that contained miRNAs and genes expression in NSCLC from Gene Expression Omnibus.

**Results:**

Our analysis results indicated that fourteen miRNAs were significantly dysregulated in NSCLC. Five of them were up-regulated (miR-9, miR-708, miR-296-3p, miR-892b, miR-140-5P) while nine were down-regulated (miR-584, miR-218, miR-30b, miR-522, miR486-5P, miR-34c-3p, miR-34b, miR-516b, miR-592). The integrating diagnosis sensitivity (SE) and specificity (SP) were 82.6% and 89.9%, respectively. We also found that 4 target genes (*p* < 0.05, fold change > 2.0) were significant correlation with the 14 discovered miRNAs, and the classifiers we built from one training set predicted the validation set with higher accuracy (SE = 0.987, SP = 0.824).

**Conclusions:**

Our results demonstrate that integrating miRNAs and target genes are valuable for identifying promising biomarkers, and provided a new insight on underlying mechanism of NSCLC. Further, our well-designed validation studies surely warrant the investigation of the role of target genes related to these 14 miRNAs in the prediction and development of NSCLC.

## INTRODUCTION

Non-small cell lung cancer (NSCLC) remains one of the leading causes of cancer death, with a high mortality rate worldwide[1, 2], accounting for over one quarter of cancer deaths in 2014 [1-3]. Recently, many studies have reported promising biomarkers for differential diagnosis of NSCLC [4 -13]. However, accurate biomarkers of NSCLC still remain largely unexplored.

Currently, the discovery of microRNAs (miRNAs), a class of small non-coding RNAs, has opened up a new perspective for cancer prediction and provides a novel approach for the initial screening of cancer, including NSCLC [14][4]. Emerging evidence has reported that miRNAs are remarkably aberrant in tumors [15-17][5-7], and may be involved in initiation and progression of NSCLC [18-20][8-10]; in addition, due to their inherent nature, miRNAs seem to remain highly stable and provide more accurate prediction factors for clinical specimens [21, 22][11, 12]. The above discovery shows that miRNAs are suitable as biomarkers for the diagnosis of NSCLC.

Unfortunately, several conflicting results are still present in independent studies [23, 24][13, 14], which are often explained by different miRNA profiling systems and platforms. Although they separately have promising value for cancer differentiation, a systematic analysis of these collected data may be essential for further exploration of the applicability of miRNAs as biomarkers for the prediction of NSCLC.

Thus, our meta-analysis answers three questions: (1) whether some of the miRNAs could differentiate tissues as NSCLC or control, (2) whether there were relationships between promising miRNAs with target genes in functional annotation and pathways, and (3) whether genes targeted by these miRNAs are associated with NSCLC initiation and progression.

## RESULTS

### Regulation and predictive value of miRNA expression in lung cancer tissue

**Figure 1:**
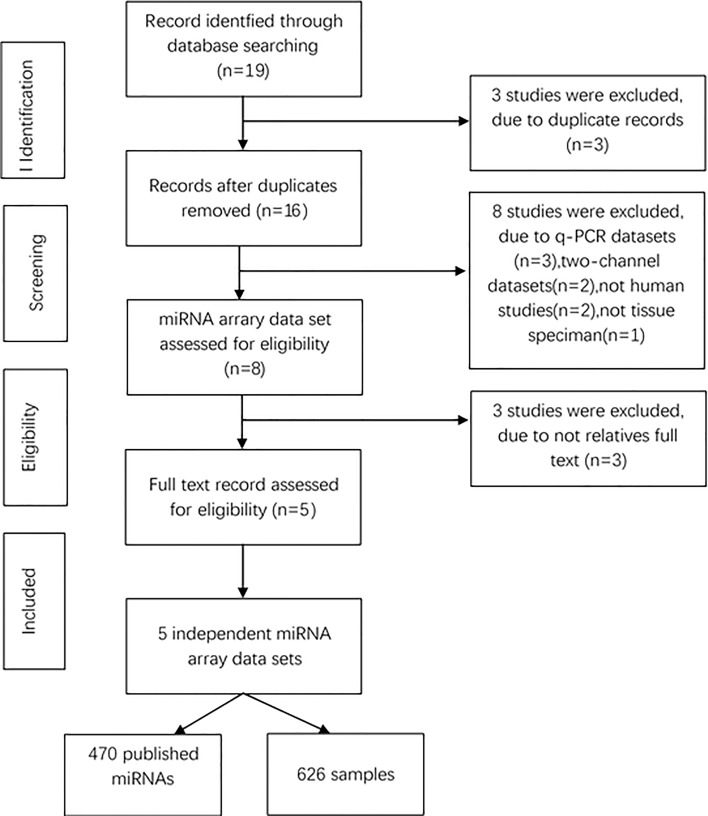
Flowchart of miRNAs studies in this meta-analysis

**Figure 2:**
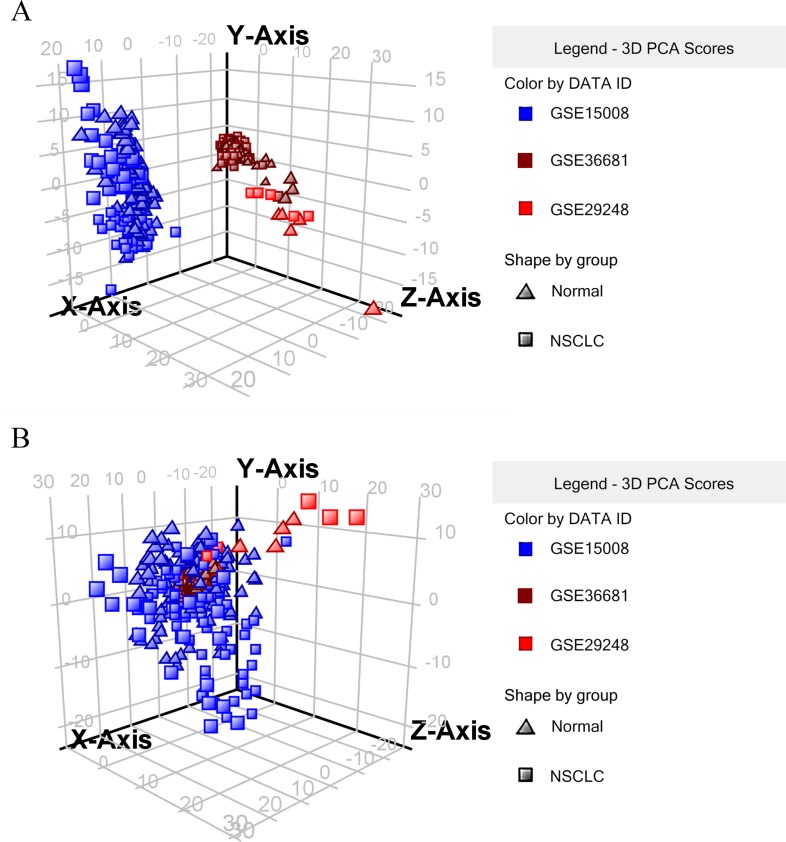
3D plot of principal components analytic scores (6 = GSE15008, 9 = GSE36681, 10 = GSE29248) **A.** raw data without normalization, **B.** with normalization and batch effect removal.

To determine whether the expression of miRNAs could be used to identify NSCLC and control cases, our initial search yielded 19 relevant data sets. After removing 3 duplicated data sets and 11 unqualified data sets (Figure 1), three primary data sets(GSE15008, GSE36681, GSE29248) as a training cohort were further examined in this meta-analysis, which was comprised of a total of 263 cancer tissue samples and 236 control tissue samples. We received another two complete sets of miRNA data (GSE51853, GSE19945) as a validation cohort, which was composed of a total of 127 tissue samples. The five lung cancer microarray data sets was used to used to analyze the miRNA expression profiles of NSCLC tissues relative to their normal controls. The characteristics of these samples are shown (Table 2, Table 4). Microarray data sets were normalized by control normalization algorithm using Agilent's GeneSpring 13.0. After normalization, batch effect was removed (Figure 2).

**Table 1:** Top 14 Significantly differentiated miRNAs in lung cancer

Gene name	*P*-value	FC(abs)	FDR	Regulation
has-miR-9	4.18E-11	2.002815	2.09E-09	up
has-miR-584	2.26E-13	1.568074	6.78E-12	down
has-miR-708	1.27E-10	1.553729	1.016E-08	up
has-mir-218	3.73E-13	1.608805	7.46E-12	down
has-miR-296-3P	5.64E-11	1.591363	1.974E-09	up
has-miR-30b	1.25E-06	1.724754	0.00003875	down
has-miR-522	1.37E-09	1.603749	3.699E-08	down
has-miR-486-5P	9.52E-07	1.759159	0.000021896	down
has-miR-34C-3P	1.49E-11	1.737232	2.831E-10	down
has-miR-892b	9.28E-13	2.103988	1.392E-11	up
has-miR-34b	1.30E-11	1.872982	1.43E-10	down
has-miR-516b	1.25E-10	1.561706	8.75E-10	down
has-mir-140-5p	1.77E-08	2.321465	5.31E-08	up
has-mir-592	4.98E-12	1.635831	4.98E-12	down

**Table 2:** all samples ' characteristics of microarray data sets used in both training and validation stage

	Training		Validation	
	Cancer (236)	Healthy (136)	Cancer (88)	Healthy (39)
**Sex**				
Male	136	87	57	22
Female	100	49	31	17
**Age, yr**				
Median	67	63	67	58
SD	8.26	10.24	8.12	10.94
Range	46 - 72	30 - 70	43 - 71	38 - 64
**Smoking history, pack-years**				
Median	32	51	45	33
SD	22.1	23.8	25.8	32.2
Range	3 - 77	5 - 56	1 - 76	5 - 65
**Tumor subtype**				
Adenocarcinoma	98		58	
Squamous	138		30	
**Tumor stage**				
Stage I	130		49	
Stage II	106		39	

**Table 3:** Top 20 Significantly differentiated target Genes in lung cancer

Gene name	Training set			Testing set		
	*P*-value	FC	FDR	*P*-value	FC	FDR
KIAA1462	2.23E-45	−3.41	1.72E-44	1.28E-20	−3.26	9.84E-20
MMD	2.58E-41	−4.46	2.17E-40	5.85E-16	−3.11	9.36E-15
CBX7	2.57E-33	−2.11	2.34E-32	5.42E-23	−2.19	1.19E-21
FAP	1.23E-27	3.07	9.47E-27	8.36E-15	3.01	1.08E-13
GPM6A	7.11E-61	−7.49	5.98E-60	2.15E-23	−4.40	1.66E-22
FAM107A	2.17E-46	−6.39	1.67E-45	2.05E-21	−3.16	1.87E-20
SEMA6A	3.49E-39	−2.98	2.69E-38	8.42E-04	−1.59	6.48E-03
THBD	1.91E-38	−3.19	1.34E-37	5.89E-18	−2.41	5.36E-17
COL1A1	9.58E-37	3.06	8.72E-36	1.75E-05	2.33	1.05E-03
STK39	1.24E-35	2.31	8.67E-35	3.62E-08	1.49	2.78E-07
PDK4	8.50E-33	−3.72	7.74E-32	3.36E-11	−2.72	2.82E-10
LIMCH1	1.49E-32	−2.52	1.35E-31	8.04E-16	−2.47	6.76E-15
OLFML1	2.60E-30	−2.60	2.18E-29	3.55E-18	−2.56	2.73E-17
TOX3	3.66E-26	3.93	3.07E-25	2.13E-09	3.50	1.79E-08
GREM1	2.28E-24	6.76	1.75E-23	5.18E-05	2.06	3.99E-04
SLC2A1	1.19E-23	2.56	8.32E-23	2.19E-06	1.76	1.61E-05
TTK	1.25E-23	3.19	9.66E-23	2.39E-11	2.61	2.17E-10
OLR1	6.33E-20	−3.22	5.76E-19	1.24E-07	−2.07	1.13E-06
SIX1	5.12E-18	2.46	3.59E-17	2.74E-08	2.03	2.11E-07
IGF2BP3	2.04E-13	2.01	1.71E-12	9.76E-11	2.66	8.88E-10

**Table 4:** Characteristics of the 9 studies in our meta-analysis of diagnosis NSCLC using microarray data sets

Database	GEO	Platform	PMID	Ethnicity	No. of miRNA/gene	NSCLC cases (No)	Healthy cases(No)	Analysis miRNA/Gene
1	GSE15008	GPL8176	21890451	China	1146	174	201	miRNA
2	GSE36681	GPL8179	22573352	USA	1146	56	56	miRNA
3	GSE29248	GPL8179	22046296	China	1146	6	6	miRNA
4	GSE51853	GPL7341	24903339	Japan	1146	80	31	miRNA
5	GSE19945	GPL9948	NA	Japan	1146	8	8	miRNA
6	GSE1987	GPL91	17258348	Israel	10610	25	9	GENE (testing)
7	GSE33532	GPL570	NA	Germany	25906	20	19	GENE (testing)
8	GSE2514	GPL8300	16314486	USA	8943	30	40	GENE (testing)
9	GSE19804	GPL570	20802022	China	54656	60	60	GENE (training)
10	GSE33532	GPL570	NA	Germany	25906	20	20	GENE (training)
11	GSE43458	GPL6244	23659968	USA	33251	40	30	GENE (training)

470 overlapped miRNAs were differentially regulated by cancer cases with a cut off p-value of 0.05 and fold change of 1.5. Meanwhile we developed an algorithm based on the Weka tool to construct miRNAs predictive models by data mining; subsequently, we selected 14 miRNAs as a new integrated training set to construct a predictive model.

The 14 miRNAs model performed stably in distinguishing between NSCLC and control cases, with a sensitivity of 82.6%, a specificity of 89.9%, a positive predictive value (PPV) of 87.5%, and a negative predictive value (NPV) of 85.8%. The AUC of the training set was 0.913. Up-regulated miRNAs (miR-9, miR-708, miR-296-3p, miR-892b, miR-140-5P) and nine down-regulated (miR-584, miR-218, miR-30b, miR-522, miR486-5P, miR-34c-3p, miR-34b, miR-516b, miR-592) in the 14 top miRNAs were significantly regulated by tumor cases (Table 1). Moreover, in validation, the 14 miRNAs model produced prediction sensitivity that increased continually and significantly: SE=88.14%, SP=91.18%, PPV=89.66%, NPV=89.86%, AUC=0.905

A study [25] reported that the target genes of multiple miRNAs play a crucial role in controlling stimulatory or inhibitory activity in tumorigenesis. Thus, the potential target genes of these miRNAs need to be identified.

### MiRNA target prediction and functional analysis

In order to identify potential miRNA target genes, we first queried the three most popular computational databases MiRBase [26], PicTar [27], and Targetscan [28] to scan target genes on the principle of mutual recognition. 1743 overlapping target genes related to the top 14 miRNAs emerged as a particular group. Then physiological pathways of target genes were analyzed using the Ingenuity Pathway Analysis (IPA) tool.

**Figure 3:**
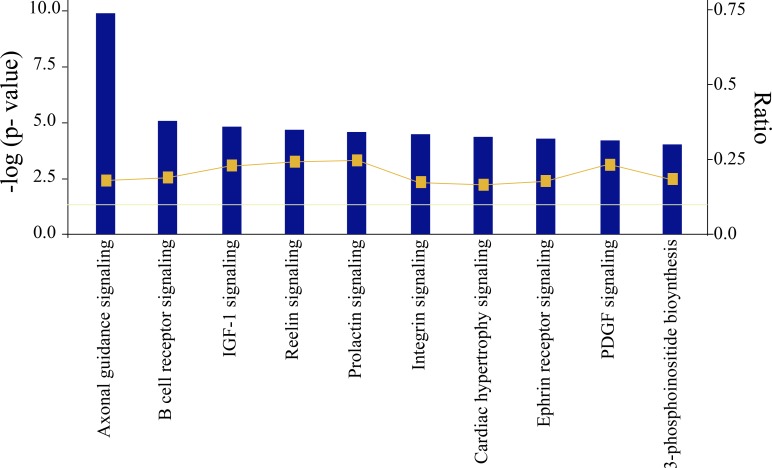
Most significant canonical pathways of putative target genes of lung cancer regulated 14 promising miRNAs (The threshold lines indicate 5% *P*-value. The bigger the -log (*p*-value) of pathway is, the more significantly the pathway is adjusted).

Interestingly, the top 10 significant pathways which are shown in Figure 3 were enriched by the 1473 genes associated with cancer initiation and progression. Among them, Axonal Guidance Signaling Pathway, Insulin-like growth factor-1(IGF-1) Signaling, Integrin Signaling Pathway, and Ephrin Receptor Signaling Pathway were highly associated with NSCLC initiation and progression. The Axonal Guidance Signaling Pathway involves 77 target genes with NSCLC, the IGF-1 Signaling Pathway involves 22, the Integrin Pathway involves 35, and Ephrin Receptor Signaling Pathway involves 31. In general, these genes were regulated by each other either directly or indirectly. Yet the underlying values for these genes associated with pathways have not been clearly illuminated. Thus, it is necessary to investigate the interaction between target genes and the 14 significant miRNAs.

### Validate the 14 significant miRNAs using gene microarray data, and explore the correlation with target genes

In this meta-analysis, the predicted performance of miRNAs was further verified by gene expression data sets. After excluding those studies according to the previous including criteria, 6 studies remained. Gene expression data sets (GSE1987, GSE43458, GSE33532 (paired), GSE2514, GSE19804, GSE33532 (unpaired)) with 7254 common genes were extracted from Gene Expression Omnibus, which included 195 NSCLC and 178 normal cases. After normalizing the raw data, controlling sample quality, correcting background, and performing log2 transformation, the miRNAs were filtered according to a *t*-test p-value cut off of 0.05 and a 1.5 fold change cut off. A Bayesian statistical analysis with 5% false discovery rate (FDR) was selected as one of three criteria for significant variable value.

**Figure 4:**
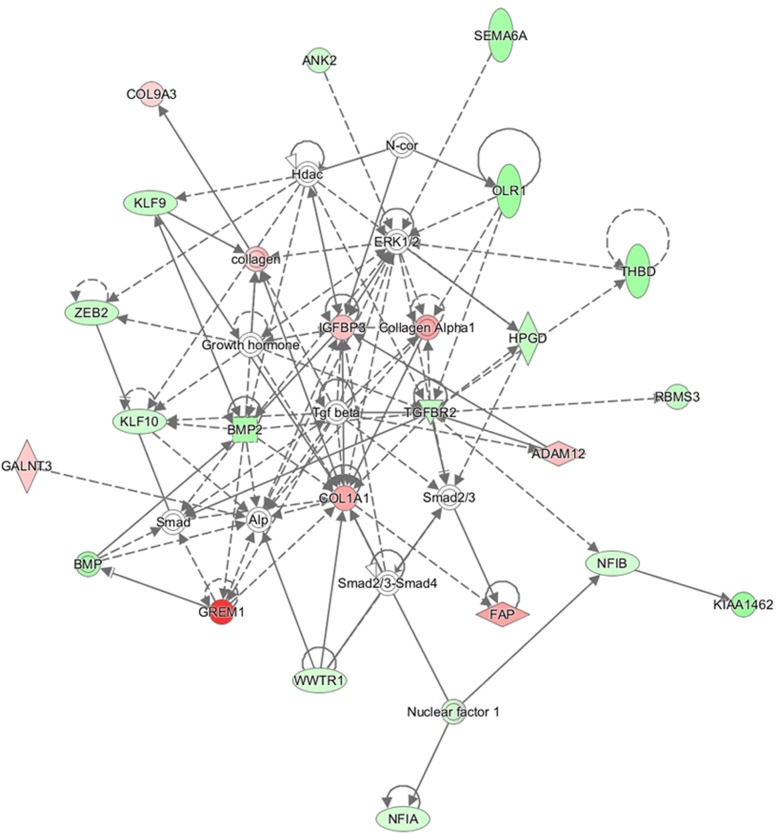
Gene network using target genes from 14 promising miRNAs The network was produced by IPA. Nodes colored in red or green indicate up-regulated and down-regulated gene, respectively.

Statistical analysis identified 1263 differentially expressed genes (*p* < 0.05, FC > 1.5) in NSCLC *versus* normal cases. Moreover, we found 900 genes with FDR < 0.05 and FC > 1.5 targeted by 14 miRNAs, in which 100 genes had an FDR < 0.05 and FC > 2.0. Among them 71 genes were down-regulated and 29 genes were up-regulated in NSCLC cases. The 100 gene list of better FDR score were uploaded into the IPA tool. A gene network was computed (Figure 4). Nodes colored in red and green indicate up-regulated and down-regulated gene respectively. We could clearly see the gene interaction between the two regulation directions. The top 20 significant genes are listed in Table 3.

To further assess the prediction abilities of gene cross-validation, we divided six data sets which were retrieved from PubMed into two sets (training set, testing set) according to sample size. The training set was composed of 3 paired specimens (GSE19804, GSE33532, GSE43458) containing a total of 230 samples respectively. The remaining 3 data sets for testing were comprised of 3 unpaired specimens (GSE1987, GSE43458, GSE2514) containing a total of 143 samples. The characteristics of these samples are shown in Table 3.

**Figure 5:**
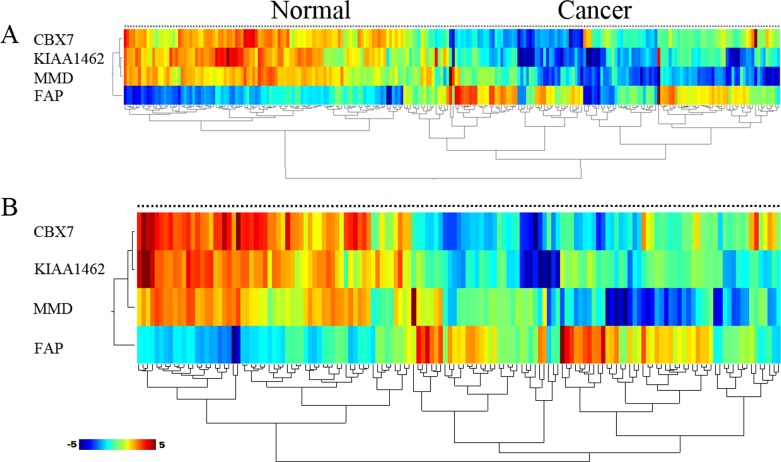
Hierarchical clustering analysis of two sets based on 4 core genes was performed using samples from (A) training set and (B) testing set The relative level of gene expression is indicted by the color scale at the bottom word “c” on the each clustering plot represent cancer sample. Word “n” on each clustering plot represent control sample.

Meanwhile we developed an algorithm based on the Weka tool to construct gene predictive models by data mining; subsequently, we selected 4 core genes as a new integrated training set to construct a predictive model. The 4 gene model performed stably in distinguishing between NSCLC and control cases, with a sensitivity of 96.7%, a specificity of 88.1%, a positive predictive value (PPV) of 89.9%, and a negative predictive value (NPV) of 96.0%. The AUC of the training set was 0.984. Moreover, in validation, the 4 genes model produced prediction sensitivity that increased continually and significantly: SE = 98.7%, SP = 82.4%, PPV = 86.1%, NPV = 98.3%, AUC = 0.933 (Table 5). In addition, hierarchical cluster analysis showed that the samples of training set and testing set were also clearly separated into 2 main classes (Figure 5). This shows that these core genes can discriminate between NSCLC cases and normal cases.

**Table 5:** Significantly differentiated target genes in lung cancer

Group(gene name)	SE	SP	PPV	NPV	AUC
Training set (MMD,CBX7, FAP,KIAA1462)	0.967	0.881	0.899	0.96	0.984
Testing set (MMD,CBX7, FAP,KIAA1462)	0.987	0.824	0.861	0.983	0.933

SE: sensitivity SP: specificity PPV: positive predictive value NPV: negative predictive value AUC: area under the curve

### Verification of microarray responses using real time QRT-PCR to verify the credibility of microarray and gene network modeling results

To verify our microarray meta-analysis results, we chose two cell lines, A549 lung adenocarcinoma cell lines and normal *lung* epithelial cells NL20, to conduct the experiments. We selected four miRNAs and all the four gene markers to perform real time quantitative PCR (QRT-PCR) in the two cell line. As illustrated in Figure 8, compared to normal control cell lines, has-miR-9, has-miR-296-3P, and the gene FAP were up-regulated whereas has-miR-522, has-miR-34b, the gene KIAA1462, the gene MMD and the gene CBX7 were down-regulated.

**Figure 6:**
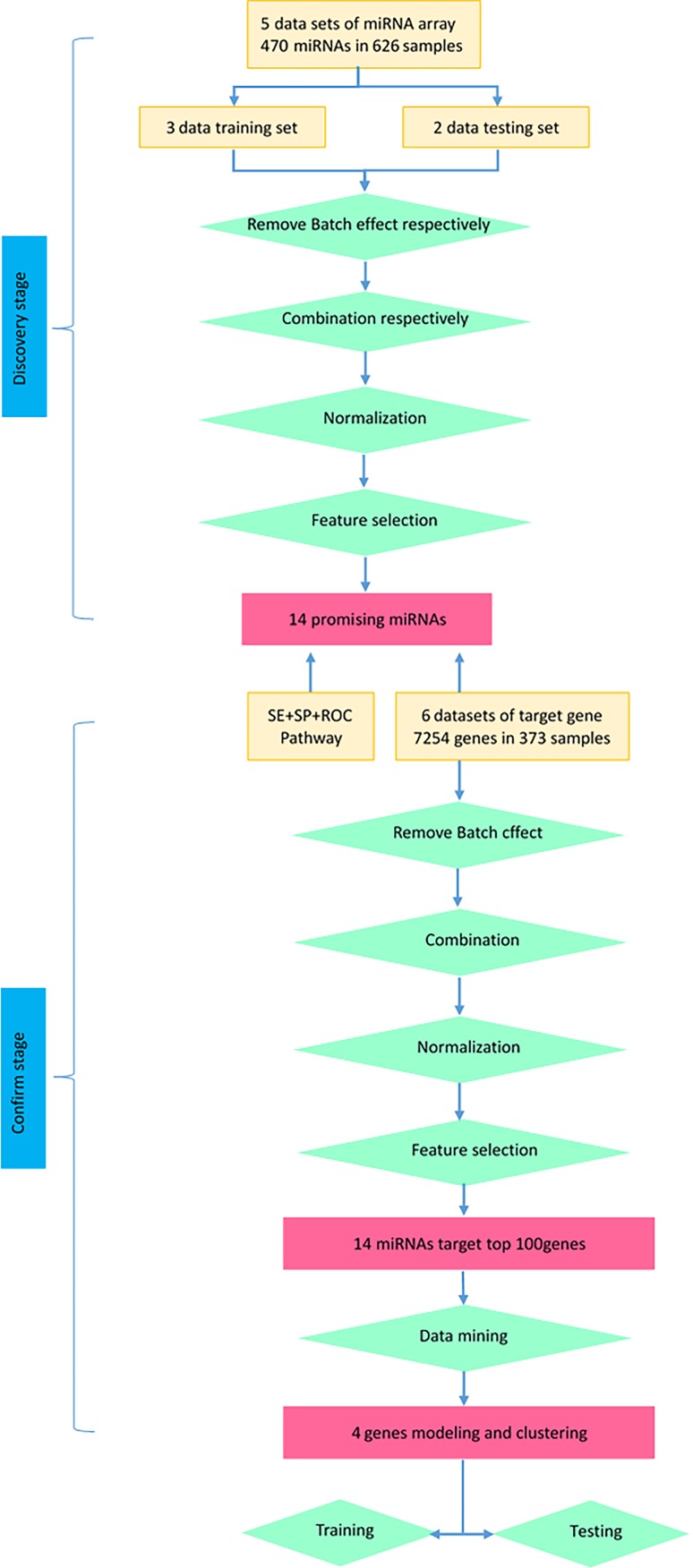
Flowchart of studies (including miRNA and target gene) in this research

**Figure 7:**
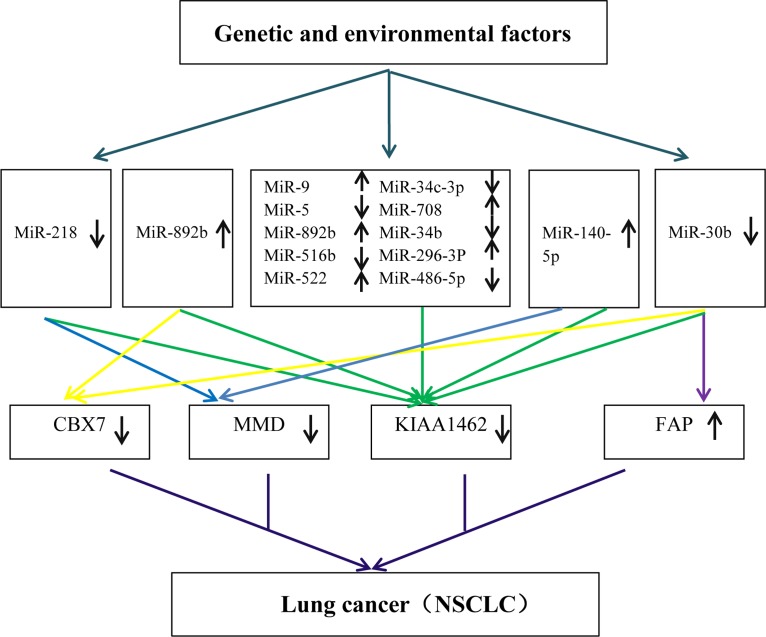
A hypothetical model to explain the molecular mechanisms of NSCLC based on enrolled data sets

**Figure 8:**
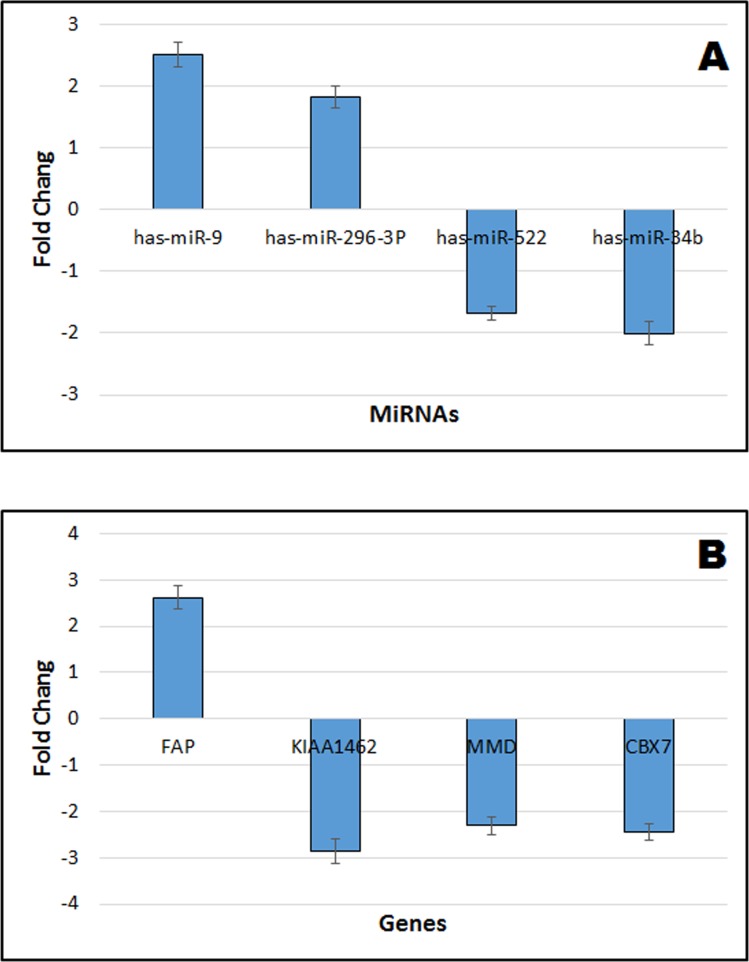
Verification of miRNA and gene expression of integrative microarray results using real time QRT-PCR **A.** Verification of 4 miRNA results. **B.** Verification of 4 gene results. The positive value indicates up-regulated fold change of lung cancer cell line A549 compared to *normal lung* epithelial cells NL20. The negative value indicates the down-regulated fold change of lung cancer cell line A549 compared to *normal lung* epithelial cells NL20. Values refer to the mean ± SD of three independent samples, each run in triplicate.

## DISCUSSION

In our study (Figure 6), we focused primary on whether promising miRNAs could act as accurate biomarkers to discriminate NSCLC from normal cases by taking advantage of miRNA array data sets. We selected 5 microarray data sets and set out to systematically identify promising miRNAs that distinguish NSCLC and control.

The top 14 miRNAs we found (has-miR-9, has-miR-584, has-miR-708, has-miR-218, has-miR-296-3p, has-miR-30b, has-miR-522, has-miR-486-5p, has-miR-34c-3p, has-miR-892b, has-miR-34b, has-miR-516b, has-miR-140-5p, has-miR-592), as a combination of miRNAs, has more accurate predicted value in distinguishing cancer cases with control cases as measured by higher sensitivity, higher specificity, and statistically significant pathways. Aberrant expressions of 12 miRNAs (miR-9, miR-584, miR-218, miR-296-3p, miR-486-5p, miR-34, miR-592, miR-30b, miR-708, miR-522, miR-516b, and miR-140-5p) were reported as potential biomarkers with diagnostic value in cancer patients, except for miR-516b and miR-892b. Aberrant expression of miR-9 contributes to tumor cell invasion, partly through directly down-regulating CBX7 protein expression [29]. MiR-140-5p significantly reduces MMD protein levels in NSCLC cells leading to inhibit cell proliferation by regulating Erk1/2 signaling [30][27]. Several miRNAs such as miR-584, miR-218, miR-486-5p, miR-34, miR-592, miR-30b, miR-522, were reported respectively to target CMBL/ PIP4K2A [31][28], Robol [29]/BMI [32, 33], ARHGAP5 [34] [31], KRAS/ PDGFR [35], BMI1 [33], CCND3 [36], Rab18 [37], PHLPP1 [38] responsible for cell proliferation migration and invasion. It is noteworthy that miR-708 and miR-296-3p were dysregulated in differential studies. Guo P et al. [36] reported that miR-708 positively influences cell proliferation, invasion, and migration by inhibiting the expression of Akt1, CCND1, EZH2, MMP2, Parp-1, and Bcl2 which are linked to an increase in death [39]. Lin KT et al. [37] mentioned that miR-708, through suppression of Rap1B, results in the reduction of integrin-mediated focal adhesion formation and the inhibition of cell migration and impaired metastasis, and that patients with high miR-708 show significantly better survival [40]. Similarly, Bai Y et al. confirmed that miR-296-3p decreases cancer cell growth by repression of EAG1 [41]. Liu X et al. [39] pointed out that miR-296-3p inhibits ICAM1 expression leading to tumor metastasis [42] Overall, this finding suggested that alterations of these genes/pathways represent meaningful risk factors in NSCLC.

In order to explore these interactions between miRNAs and target genes, we decided to perform a pathway analysis using the list of overlapped target genes referenced by the three computational databases. The top 10 significant pathways enriched 1473 genes associated with cancer initiation and progression.

In our following study of target genes, we took advantage of statistical computer tools to mine some available data for target genes, and then subsequently hold overlapping genes. We found that over half of the target genes with better FDR and higher FC were involved in NSCLC. The gene networks showed that many of these genes related to NSCLC, and interacted with each other.

To seek genes offering greater sensitivity and specificity, a statistical model based on six gene data sets was built. Finally, we selected the 4-gene index (KIAA, MMD, CBX7, and FAP) as a novel biomarker for diagnostic prediction of NSCLC. The index achieved 96.7% sensitivity, 88.1% specificity, 89.9% PPV, and 96.0% NPV in the training set, and with higher significance in testing set (SE = 98.7%, SP = 82.4%, PPV = 86.1%, NPV = 98.3%). Emerging reports [27, 40, 41] showed that MMD, CBX7, FAP played an important role in the proliferation of lung cancer [30, 43, 44]. It is noteworthy that the gene KIAA has never been reported as related to cancer, serving only as a risk factor in coronary artery disease [45][42]. Our study showed KIAA was frequently directly or indirectly associated with the 14 promising miRNAs in NSCLC. Therefore, based on both miRNAs and target genes level, we generated a hypothetical model that can explain genetic and environmental factors that trigger NSCLC (Figure 7). Genetic and environmental factors could affect the expression of miR-9, miR-584, miR-708, miR-218, miR-296-3p, miR-30b, miR-522, miR-486-5p, miR-34c-3p, miR-892b, miR-34b, miR-516b, miR-140, miR-592, then that of KIAA, MMD, CBX7, and FAP, and through interaction finally result in tumorigenesis. As illustrated in our experiment, has-miR-9, has-miR-296-3P, and the gene FAP were up-regulated whereas has-miR-522, has-miR-34b, the gene KIAA1462, the gene MMD and the gene CBX7 were down-regulated. The QRT-PCR results were consistent with the microarray meta-analysis results.

Overall, our results not only demonstrate that combining miRNAs and target genes improves our ability to identify promising biomarkers, but it also contributes to greater insight on new potential mechanisms and functions for predicting NSCLC.

Although we tried to avoid bias in our study, certain limitations still need to be considered while interpreting the result of our study. More research with experimental validation is clearly needed in order to find promising miRNAs and target genes using microarray platform to real-time RT-PCR assays, allowing broader accession and utilization in future clinical application. Third, further work is needed to investigate the relationship between miRNAs and genes.

Despite the above limitations, our study is the first meta-analysis to predict NSCLC from microarray data sets at both miRNA and gene level. This study also avoids distinguishing expression patterns in promising target genes that contradict those of the miRNAs. Detecting NSCLC using miRNAs and core genes still needs further validation as well.

## MATERIALS AND METHODS

### Search strategy, eligibility and data extraction

Microarray data sets were extracted from NCBI and Gene Expression Omnibus by means of the MESH terms ‘lung cancer/lung neoplasm/NSCLC’ and ‘microRNAs/miRNA’, in combination with the keyword ‘lung tumor/lung neoplasm/NSCLC’ and ‘gene expression/target gene’, without restriction of language or publication.

Three reviewers (Ling Hu, Junmei Ai, and Hui Long) independently extracted the following data from all eligible studies. Eligible data sets had to meet the following criteria: all sample data sets (i) were from humans, (ii) focused on the diagnostic potential of miRNAs/genes for LC tissue, (iii) included microRNA array, (iv) came from raw data rather than matrix data/normalized data, and (v) were part of studies with included false discovery rate (FDR) and fold-change (FC) calculations. All data sets used in this study are summarized in Table 4.

### MiRNAs and genes microarray data processing

The scale of miRNAs/genes expression in microarray data sets was consistently different due to different platforms and different batches [46][15]. All statistical data sets were normalized and standardized to be approximately equal in scale and normally distributed. There is general agreement on the normalization of single miRNA/gene expression using the median value of expression of all miRNAs/genes of each data set [47, 48][16, 17], and the expression of each case in each data set was compared with the respective control samples. We combined log_2_ transformed data sets from different platforms into three: the miRNA data set, a paired gene data set, and an unpaired gene data set. 5% FDR in Bayesian statistical analysis was used to find statistically significant differential miRNAs between cancer and control cases.

### Verification of miRNA and gene expression of integrative microarray results using real time QRT-PCR

### Cell culture

A549 lung adenocarcinoma cell lines and *normal lung* epithelial cells (NL20) were purchased from the American Type Culture Collection (ATCC). The cells were cultured in minimum essential medium, Dulbecco's modified Eagle's medium (DMEM), and Ham's F12 medium supplemented with 10% fetal bovine serum (FBS) (Sigma Chemical Co., St. Louis, USA), penicillin (100 U/ mL) and streptomycin (100 μg/ mL) as antibiotics in a humidified atmosphere of 5% CO_2_ at 37°C.

### RNA extraction

Total RNA was extracted using Qiagen miRNeasy kit (Qiagen, Valencia, CA) according to the manufacturer's protocol. In brief, the cell pellet was mixed with QIAzol Lysis Reagent and chloroform. After centrifugation at 12,000g at 4°C for 15 min, the aqueous phase was transferred into another tube, and 1.5 volumes of absolute ethanol were added. The mixture was then applied to miRNeasy Mini kit columns, following by washing with RWT and RPE buffers. The RNAs were finally eluted in 40 μl of RNase-free water.

### Quantitative RT-PCR

MiRNAs and genes were measured using Taqman miRNA assay kits (Applied Biosystems, USA) according to the manufacturer's protocol. Briefly, about RNA was reverse transcribed with a TaqMan Reverse Transcription Kit (Applied Biosystems, USA). Expression levels of miRNAs and genes were quantified in triplicate by qRT-PCR using human TaqMan Assay Kits (Applied Biosystems, USA) on the ABI 7500 thermocycler (Applied Biosystems) according to the manufacturer's protocol. The expression value of miRNAs were normalized against an internal control (U6 RNA) and expression value of genes (mRNAs) were normalized using the internal control GAPDH.

## Abbreviations

NSCLC: non-small cell lung cancer
SE: sensitivity
SP: specificity
PPV: positive predictive value
NPV: negative predictive value
ROC curve: receiver operating characteristic curve
AUC: area under ROC curve
FDR: False discovery rate
FC: fold change
GO: Gene Expression Omnibus
